# Preventive Effects of *Crataegus monogyna* Fruit Extract on Oxidative Damage and Inflammatory Response in a Rat Model of Acetic Acid‐Induced Ulcerative Colitis

**DOI:** 10.1002/fsn3.72074

**Published:** 2026-07-05

**Authors:** Soumaya Wahabi, Dhekra Grami, Khaoula Gharbi, Slimen Selmi, Hichem Sebai

**Affiliations:** ^1^ Higher Institute of Biotechnology of Beja, LR: Functional Physiology and Valorization of Bio‐Resources University of Jendouba Beja Tunisia

**Keywords:** *Crataegus monogyna*, inflammation, oxidative stress, ulcerative colitis, wistar

## Abstract

BackgroundUlcerative colitis (UC) is a chronic inflammatory bowel disease associated with oxidative stress and inflammation. Natural products may offer protective effects, though their mechanisms remain unclear. Hawthorn fruit ( *Crataegus monogyna* ) is known for its antioxidant and anti‐inflammatory properties, providing a rationale to explore its potential against UC ObjectiveTo evaluate the protective effects and mechanisms of *Crataegus monogyna* fruit extract (CMFE) in acetic acid‐induced UC in rats. MethodsSix groups of Wistar rats were used: control, acetic acid (AA), AA + CMFE (100, 200, 400 mg/kg, p.o.), and AA + sulfasalazine (100 mg/kg, p.o.). Treatments lasted 10 days, followed by AA rectal infusion (300 mg/kg). Colonic damage was assessed macroscopically and histologically. Oxidative stress markers (MDA, thiols, SOD, CAT, GPx) were measured in colon homogenates, while CRP, ALP, iron, and calcium levels were determined in plasma. ResultsCMFE pretreatment significantly reduced macroscopic and microscopic damage, protecting the colonic mucosa from AA‐induced lesions. AA increased MDA and decreased SH‐group as well as antioxidant enzyme activities, whereas CMFE restored these parameters. CMFE also normalized CRP, ALP, iron, and calcium levels, counteracting AA‐induced inflammatory and metabolic disturbances. ConclusionCMFE shows strong protective effects against AA‐induced colitis by modulating oxidative stress and inflammation. These findings support its potential use in green medicine as a natural therapeutic agent for inflammatory bowel diseases.

## Graphical abstract

*Crataegus monogyna* fruit extract attenuated oxidative stress and inflammatory responses in acetic acid‐induced ulcerative colitis in rats. Treatment improved antioxidant status, reduced lipid peroxidation, and alleviated histopathological damage, demonstrating its protective effect against ulcerative colitis.

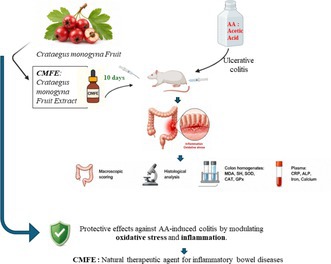

## 1 Introduction

Inflammatory bowel disease (IBD) is a term used to describe persistent, non‐specific inflammations that mostly affect the gastrointestinal system and are caused by autoimmune illnesses (Kong et al. 2024). IBD often manifests in two primary forms: ulcerative colitis (UC) and Crohn's disease (CD). While the precise cause of IBD is still unknown, a number of factors, such as genetic predisposition, gut microbiota, environmental factors, and mental health disorders, have been linked to disease processes (Xu et al. 2022). Affecting 5 million people worldwide, ulcerative colitis (UC) is a chronic, recurrent inflammation of the gastrointestinal tract, clinically manifested by abdominal pain, chronic diarrhea, rectal bleeding and weight loss. However, existing treatments are ineffective, and UC patients have a lower life expectancy and a higher risk of colorectal cancer (Wangchuk et al. 2024). UC mostly affects the colon, resulting in a continuous pattern of superficial mucosal inflammation that spreads proximally (Chang 2020). Furthermore, UC is characterized by dysfunction or premature death of epithelial cells, a reduction in the mucus layer and dysregulation of underlying intestinal immunity, which compromises barrier protection (Martini et al. 2017). Impaired barrier integrity is not only clinically evident in IBD, but also contributes to a variety of digestive tract diseases (Nalle and Turner 2015). As a result, treating IBD calls for a multifaceted therapeutic strategy that includes medication designed to lower inflammation and stop immune system abnormalities. Among the medications given to treat digestive tract issues include biologics immunosuppressants and anti‐inflammatory medicines (Lee et al. 2024). Treatment with nutrition is a crucial component of managing symptoms and has the potential to improve general health. In certain instances, surgery may be needed to eliminate damaged parts of the digestive tract. However, drugs may cause long‐term adverse consequences, such as an elevated risk of infections and some types of cancer (Beaugerie et al. 2020; Stallmach et al. 2010). For this reason, research on complementary and alternative medicines, such as dietary modifications, probiotic and herbal supplementation, and the creation of safer, more focused treatments, is still ongoing (Ma et al. 2023; Parian and Limketkai 2016). *Crataegus monogyna* is a widespread species in Tunisia but is rarely used. *C. monogyna* (hawthorn) is a useful therapeutic plant from the *Rosaceae* family (Paun et al. 2024). It is used in traditional medicine to treat digestive, microbial and cardiac problems, thanks to its antispasmodic, cardiotonic, antiatherosclerotic and hypotensive properties, as well as diabetic disorders (Fakchich and Elachouri 2021). *C. monogyna* is a great source of bioactive compounds, especially polyphenols such as isoquercitrin, vitexin, epicatechin, procyanidins and chlorogenic acid, but also triterpenic acids such as ursolic acid and oleanolic acid, carboxylic acids and minerals (Edwards et al. 2012; Nabavi et al. 2015; Radi et al. 2023). Through the control of inflammatory responses and antioxidant qualities in a rat model of colitis, this study sought to assess the preventive coloprotective effect of *Crataegus monogyna* fruits extract (CMFE) on AA‐induced ulcerative colitis.

## 2 Materials and Methods

### 2.1 Plant Collection and Extract Preparation

During October and November of 2023, *Crataegus monogyna* fruits were gathered from Beja region (N‐W, Tunisia), and identified by Dr. Chokri Hafsi, botanical coordinator at the Institute of Biotechnology of Béja, University of Jendouba. The fruit was then vigorously smashed in a conventional metallic mortar and subsequently in an electric blender after being dried at 40°C for 5 days with air circulation. The final extraction yield was 40.93% ± 1.57% (*n* = 3), calculated as the ratio between the mass of the dry extract obtained after lyophilization and the initial dry mass of the plant material. The powder was added to boiling bi‐distilled water (70°C) and agitated for 1 h to create CMFE. A Buchner funnel and Whatman No. 1 filter paper were used to filter the aqueous extracts. A freeze dryer (Refrigerated Vapor Trap RVT450) was used to dry the filtrates for 48 h after they had been rapidly frozen at−40°C. The aqueous extract was chosen based on its traditional use and high phenolic content. Doses of 100, 200, and 400 mg/kg were selected based on preliminary dose–response studies and previous research on Crataegus species in gastrointestinal models (Wahabi et al. 2023).

### 2.2 Plant Extract Characterization of Phenolic Compounds by LC–ESI–MS

100 mg of CMFE extract were dissolved in 100 mL of 10% methanol and filtered, and then 1 mL was transferred into LC–MS vials. An opposite‐phase column (Pursuit XRs ULTRA 2.8, C18, 100 × 2 mm, Agilent Technologies, UK) was used to carry out HPLC investigations.

20 mL of the prepared samples were injected at a column temperature set at 30° *C. Mobile* phases consisted of 0.1% formic acid in water (A) and 0.1% formic acid in methanol (B). A gradient program was used for isolation at a flow rate of 1 mL min −1. Mobile phases consisted of an initial composition of 100% solvent A, with a gradient of 100% fsolvent B over 20 min, held at 100% solvent B for 5 min and 100% solvent A for 25 min. The drying gas flow rate was 1 mL min −1 at 320°C. MS was operated in the positive ion mode in a mass range of 100–2000 m/z. High‐resolution mass spectral data were obtained on a Thermo Instruments ESI‐MS system (LTQ XL/LTQ Orbitrap Discovery, UK) connected to a Thermo Instruments HPLC system (Accela PDA Detector, Accela PDA Autosampler, and Accela Pump) (Jdir et al. 2017).

### 2.3 Animals and Treatment

Thirty adults male *Wistar* rats, weighing 220 ± 20 g, housing five per cage, and 15 weeks of age, were used in the experiments. They were acquired from SIPHAT (Tunisia). Based on the NIH recommendation, animals were used in compliance with Tunis University's local ethics committee regarding the use and care of animals. The protocol has been approved by the “Pasteur Institute of Tunis's” “Comited’ Ethique Bio‐medicale (CEBM)” (JORT472001). They were kept in an animal house with a 12‐h light–dark cycle and a controlled temperature of 22°C ± 2°C. They were fed the standard diet and water as needed. There were six groups of rats, each consisting of five rats. As a control, Group 1 was given 5 mL/kg, b.w., p.o. of physiological solution (NaCl, 0.9%, p.o.). As a UC group, Group 2 was given 5 mL/kg, b.w., p.o. of physiological solution (NaCl, 0.9%, p.o.). Groups 3, 4, and 5 were pre‐treated with different doses of CMFE (100, 200, and 400 mg/kg, b.w., p.o.). Lastly, group 6 acted as a standard group and received a pretreatment of 100 mg/kg, b.w. of sulfasalazine, a standard drug. Oral gavage was used to provide all of these therapies orally for 10 days (Thippeswamy et al. 2011).

### 2.4 Induction of Ulcerative Colitis in Rats

Overnight fasting was maintained for all of the animals, on the tenth day. Using a polyethylene tube that was introduced through the rectum into the colon up to a distance of 8 cm, acetic acid (AA) (3%, v/v, 5 mL/kg, b.w.) was infused for 30 s to induce UC in all groups except the controls. 25 h later, the animals were euthanised by decapitation after deep anesthesia, and the colon was quickly removed and inspected under a microscope (Jabri et al. 2021; Thippeswamy et al. 2011). According to the review by Randhawa et al. (2014), acetic acid–induced colitis is a well‐established, easily inducible chemical model that causes reproducible mucosal injury, neutrophil infiltration, and oxidative imbalance similar to key features of human ulcerative colitis. This rapid and consistent model is therefore particularly suitable for examining acute inflammatory and oxidative stress pathways and for the initial screening of potential therapeutic agents such as CMFE. Blood samples were collected by cardiac puncture under anesthesia immediately after euthanasia. Plasma was separated by centrifugation and stored at−80°C until biochemical analyses.

### 2.5 Assessments of Ulcerative Colitis

Fecal residues were eliminated by removing the distal sections of the colon and cleaning them with physiological saline. To investigate and assess macroscopic colon inflammation, colons were sliced longitudinally. Miller et al. used an arbitrary scale with a range of 0 to 4 to determine inflammation ratings. In this manner: 1 (mucosal erythema alone), 2 (mild mucosal edema, slight bleeding ulcers or erosions), 3 (moderate edema, slight bleeding ulcers or erosions), and 4 (severe ulceration, edema, and tissue necrosis) are the first, second, third, and fourth, respectively (Thippeswamy et al. 2011). The “% protection” in Table 2 was calculated based on the reduction in gross lesion score relative to the UC group using the following formula: % Protection = [(Score UC—Score Treated)/Score UC] × 100.

### 2.6 Microscopic Evaluation of Ulcerative Colitis

Following sacrifice, samples from each treatment group were used in a histopathological investigation. Colon tissues were embedded in paraffin after being treated in 10% paraformaldehyde. Samples were then deparaffinized, rehydrated, and cut into 5 mm sections. Hematoxylin and eosin staining was applied to these slices in accordance with accepted histology practices. The histological assessment of colonic sections was based on a semiquantitative scoring system, considering five features: mucosal architecture, muscle thickness, cellular infiltration, crypt abscesses, and goblet cell mucus depletion. Each parameter was defined and scored using a standardized semi‐quantitative scale, and the total histological score was calculated as the sum of the individual subscores. The evaluation was performed independently to reduce bias.

### 2.7 Plasma Analysis

Total cholesterol (TC), high‐density cholesterol (HDL‐C), triglycerides (TG), iron (Fe), calcium (Ca), and inflammatory marker assays such as C‐reactive protein (CRP) and alkaline phosphatase (ALP) activity were evaluated in addition to glycemia. A SELECTRA PRO XL automatic biochemical analyzer and the appropriate commercial kits (ELI Tech Group Clinical system SAS, Tunisia) were used to quantify the results of plasma biochemical analysis.

### 2.8 Colon Mucosal Homogenate Preparation

The colon mucosa was immediately collected and homogenized using a T‐18 digital Ultra‐Turrax homogenizer in Tris‐buffered saline (TBS) buffer (50 mM, pH 7.6). The homogenates were then centrifuged at 3000 g for 15 min at 4°C. The resulting supernatants were used to determine malondialdehyde (MDA) and protein levels, as well as the activities of antioxidant enzymes, including superoxide dismutase (SOD), catalase (CAT), and glutathione peroxidase (GPx).

### 2.9 Biochemical Assays

#### 2.9.1 Protein Concentrations

Protein concentrations were determined according to the method of Bradford (Bradford 1976).

#### 2.9.2 Lipid Peroxidation Measurement

For MDA measurement via the double heating method (Draper and Hadley 1990), the peroxidation of the lipids was ascertained. To summarize, a BHT‐TCA solution containing 1% BHT (w/v) dissolved in 20% TCA (w/v) was combined with aliquots of colon mucosa homogenates and centrifuged at 1000 g for 5 min at 4°C. After mixing the supernatant with.5 N HCl and 120 mM TBA in 26 mMTris, it was heated for 10 min at 80°C. After cooling, the resultant chromophore's absorbance was measured at 532 nm. An extinction coefficient of 1.56 × 105 M^_1^ cm^_1^ for the MDA‐TBA complex was used to calculate the MDA levels.

#### 2.9.3 Antioxidant Enzyme Activities Assays

##### 2.9.3.1 Superoxide Dismutase Activity Assay

Modified epinephrine assays (Kakkar et al. 1984) were used to determine the SOD activity. The superoxide anion competes with epinephrine auto‐oxidation to adrenochrome at alkaline pH, reducing the production of adrenochrome. The amount of extract that reduces the rate of adrenochrome production by 50% is known as a SOD unit. A 2 mL reaction mixture including 10 μL bovine catalase (.4 U/μL), 20 μL epinephrine (5 mg/mL), and 62.5 mM sodium carbonate/bicarbonate buffer pH 10.2 was mixed with the enzyme extract. Changes in absorbance were noted at 480 nm.

##### 2.9.3.2 Catalase Activity Assay

The CAT activity was evaluated by measuring the initial rate of hydrogen peroxide (H_2_O_2_) disappearance at 240 nm (Aebi 1974). The reaction mixture contained 33 mM H_2_O_2_ in 50 mM phosphate buffer at pH 7.0, and the activity of CAT was calculated using the extinction coefficient of 40 mM^_1^ cm^_1^ for H_2_O_2_.

##### 2.9.3.3 Glutathione Peroxidase Activity Assay

Using the Floh'e and Gunzler approach, the GPx activity was measured (Flohé and Günzler 1984). Simply, 1 mL of reaction mixture containing 0.2 mL colon homogenates supernatant, 0.2 mL (0.1 M) phosphate buffer pH 7.4, 0.2 mL GSH (4 mM), and 0.4 mL H_2_O_2_ (5 mM) was incubated at 37°C for 1 min and the reaction was stopped by addition of 0.5 mL TCA (5%, w/v). After centrifugation at 1500 g for 5 min, aliquot (0.2 mL) of the supernatant was combined with 0.5 mL of 0.1 M phosphate buffer pH 7.4 and 0.5 mL of DTNB (10 mM) and the absorbance was read at 412 nm. The GPx activity was measured in nanomolar GSH consumption per minute per protein milligram.

#### 2.9.4 Total Thiol Groups Measurement

Colon homogenates were added to 0.25 M Base/Tris and 20 mM ethylene diamine tetraacetic acid, pH = 8.2 (Hu 1994). The mixture was vortexed and its absorbance was determined at 412 nm. The first value was A1. After that, 10 mM 5,5‐dithiobis (2 nitrobenzoic acid) (DTNB) was added. After incubation for 15 min, a new value A2 was determined. The white tube of DTNB contained only DTNB and buffer; its absorbance value was noted as B. We calculated the concentration of thiol groups per tube by using this expression: (A2‐A1‐B) × 1.57 mM.ost hoc test.

### 2.10 Statistical Analysis

The standard error of the mean, or mean ± SEM, was used to express all of the results' values. One‐way analysis of variance (ANOVA) followed by Tukey's post hoc test was used to compare the groups using the Graph Pad Prism statistical program, Version 8.01 (Graph Pad program Inc., La Jolla, CA, USA). *p*‐values were deemed significant if they were less than 0.05.

## 3 Results

### 3.1 LC–ESI–MS Analysis

**FIGURE 1:**
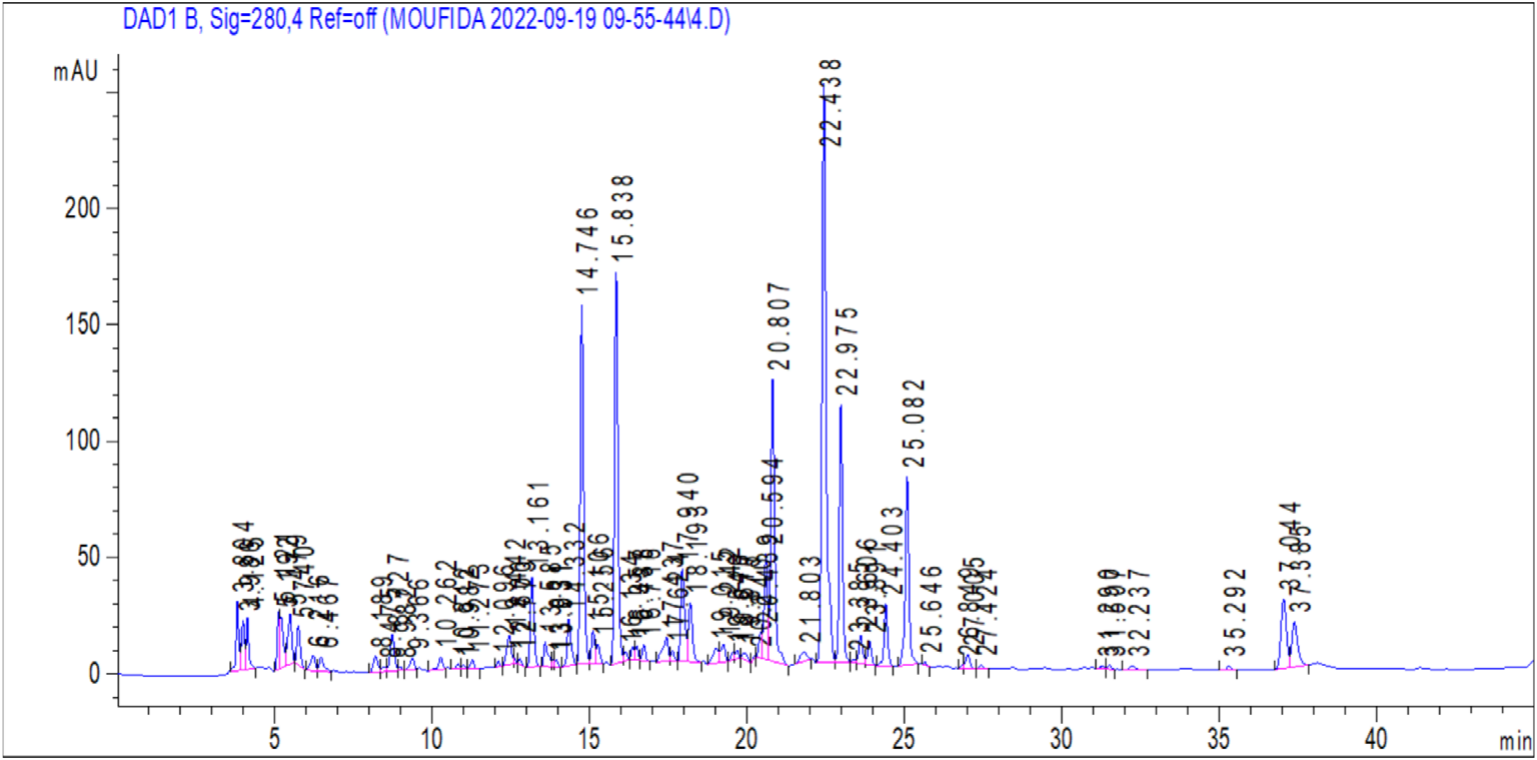
Chromatographic profiles of CMFE.

The LC–MS technique allowed us to identify six phenolic compounds in CMFE. These compounds belong to two main classes of polyphenols: phenolic acids and flavonoids. Among the phenolic acids found in CMFE, we identified syringic acid, ferulic acid, and sinapic acid. Additionally, three flavonoids were detected in the CMFE, namely catechin, myricetin, and quercetin (Figure 1 and Table 1) (Wahabi et al. 2024).

**TABLE 1:** Identification of phenolic compounds in CMFE through the LC–MS technique.

NO	Compounds	Molecular formula	Molecular mass	[M‐H] − m/z	Retention time (min)	CMFE
1	Catechin	C_15_H_14_O_6_	290	289	12,561	5.26
2	Syringic Acid	C_9_H_10_O_5_	198	197	14,609	20.80
3	Sinapic Acid	C_11_H_12_O_5_	224	169	18,840	3.90
4	Ferulic Acid	C_10_H_10_O_4_	194	193	19,206	18.39
5	Myricetin	C_15_H_10_O_8_	318	317	22,546	40.14
6	Quercetin	C_15_H_10_O_7_	302	301	26,161	11.63

### 3.2 Macroscopical Assessment of Colonic Lesions

**FIGURE 2:**
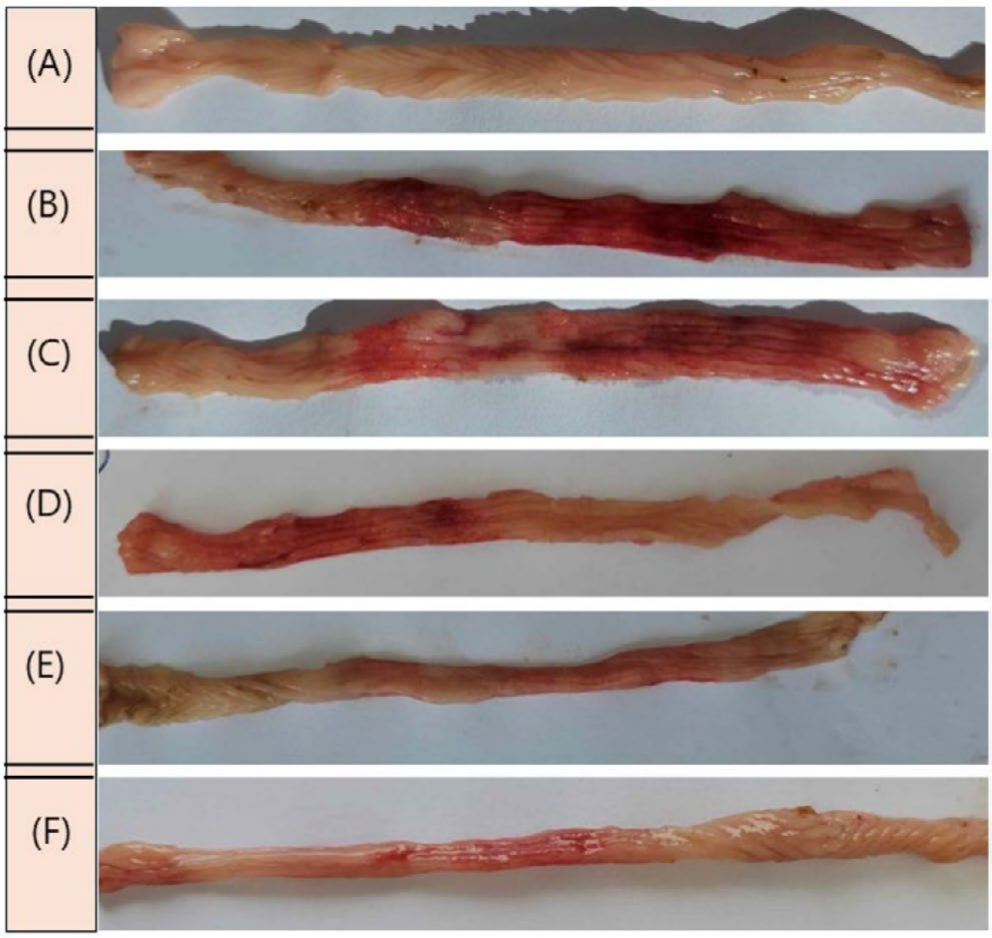
Impact of *Crataegus monogyna* fruits extract (CMFE) and sulfasalazine (SULF) on morphological variations in the colon as a result of acetic acid (AA) exposure. Animals were pre‐treated with various doses of the CMFE (100, 200 and 400 mg kg^−1^, b.w., p. o.) and SULF (100 mg kg^−1^, b.w., p. o.) or distilled water, and challenged with a single anal administration of AA (300 mg kg^−1^, b.w.) (3%, v/v, 5 mL kg^−1^, b.w.) or NaCl (0.9%, 5 mL kg^−1^, b.w.) for 24 h. (A) Control group, (B) AA group, (C) AA + CMFE (100 mg/kg, b.w., p. o.), (D) AA + CMFE (200 mg/kg, b.w., p. o.), (E) AA + CMFE (400 mg/kg, b.w., p. o.), (F) AA + SULF.

In our initial investigation, we proved that an intrarectal giving of 3% AA to animals led to a marked reduction in colonic length and an overweight colon, hence increasing the wet weight/length ratio (Table 2). Additionally, AA‐injected rats' colons displayed significant black hemorrhagic lesions, mucosal necrosis, erosion, hyperemia, and oedema upon macroscopic examination. No changes in pathology were found in the control groups (Figure 2). However, pretreatment with sulfasalazine (100 mg/kg, b.w.) and CMFE (400 mg/kg, b.w.) significantly protected the colonic mucosa from all AA‐induced lesions (Figure 2).

**TABLE 2:** Effects of sulfasalazine (SULF) and *Crataegus monogyna* fruit extract (CMFE) on acetic acid (AA)‐induced alterations in colon weight to length ratio and gross lesion score. The animals were challenged with a single anal dosage of AA (300 mg kg^−1^, b.w.) (3%, v/v, 5 mL kg^−1^, b.w.) or NaCl (0.9%, 5 mL kg^−1^, b.w.) for 24 h after being pre‐treated with varying doses of CMFE (100, 200, and 400 mg kg^−1^, b.w., p.o.) and SULF (100 mg kg^−1^, b.w., p.o.) or distilled water.

Treatment	Wet colon weight/length (mg cm −1)	Gross lesion score (Median, range)	% protection
Control	83.56 ± 7.99	0 (0–0)	—
UC	167.06 ± 17.83***	4 (4–4) *	—
UC + CMFE 100	133.80 ± 13.18^###^	4 (3–4)	0%
UC + CMFE 200	126.58 ± 12.94^###^	2 (2–3)	50%
UC + CMFE 400	111.00 ± 33.95^###^	1 (1–2)^##^	75%
UC + SULF	93.03 ± 12.13^###^	1 (1–1)^###^	75%

*Note:* Data are expressed as mean ± SEM (*n* = 5) for wet colon weight/length and as median (range) for gross lesion scores. Statistical analysis was performed using one‐way ANOVA followed by Tukey's test for parametric data and Kruskal–Wallis followed by Dunn's post hoc test for non‐parametric data.

* *p* < 0.05.

*** *p* < 0.001 vs. control group.

^##^ *p* < 0.01.

^###^ *p* < 0.001 vs. UC group.

### 3.3 Impact of CMFE and AA on Plasma Metabolic and Biochemical Markers

#### 3.3.1 Calcium and Fer Content

We also looked at how intracellular mediators, including plasma levels of calcium and Fe (free iron), were affected by acetic acid and CMFE (Table 3). Acetic acid significantly raised the calcium and free iron levels in the UC group in comparison to the negative control group. However, CMFE and sulfasalazine significantly and dose‐dependently corrected all the intracellular mediator dysregulations associated with AA intoxication.

**TABLE 3:** Effects of *Crataegus monogyna* fruits extract (CMFE) and sulfasalazine (SULF) on acetic acid (AA)‐induced changes in plasma free iron and calcium levels. Animals were pre‐treated with various doses of the CMFE (100, 200 and 400 mg kg^−1^, b.w., p.o.) and SULF (100 mg kg^−1^, b.w., p.o.) or distilled water, and challenged with a single anal administration of AA (300 mg kg^−1^, b.w.) (3%, v/v, 5 mL kg^−1^, b.w.) or NaCl (0.9%, 5 mL kg^−1^, b.w.) for 24 h.

Treatment	Fe (μmol/L)	Calcium (mmol/L)
Control	3.30 ± 0.05	2.48 ± 0.016
UC	6.53 ± 0.52***	5.15 ± 0.47***
UC + CMFE 100	5.50 ± 0.06^##^	4.11 ± 0.42^###^
UC + CMFE 200	4.51 ± 0.26^###^	3.50 ± 0.06^###^
UC + CMFE 400	3.15 ± 0.45^###^	2.53 ± O.13^###^
UC+ SULF	3.41 ± 0.43^###^	2.46 ± 0.14^###^

*Note:* Data are expressed as means ± SEM, with different groups compared by one‐way ANOVA followed by Tukey's post hoc test (*n* = 5).

*** *p* < 0.001 compared to control group.

^##^ *p* < 0.01.

^###^ *p* < 0.001 compared to AA group.

#### 3.3.2 Glycemia and Lipid Metabolism Markers

We also examined the protective impact of CMFE on lipid metabolism and glucose imbalances caused by AA. Our findings revealed a significant rise in glucose levels, as well as a slight though not statistically significant increase in TG and TC following AA exposure in the UC group. However, pretreatment with CMFE effectively reduced these metabolic disruptions in a dose‐dependent manner. Sulfasalazine, used as a reference drug, exhibited similar outcomes (Table 4).

**TABLE 4:** Effects of *Crataegus monogyna* fruits extract (CMFE) and Sulfasalazine (SULF) on acetic acid (AA)‐induced changes in glycaemia and plasma lipid profile. Animals were pre‐treated with various doses of the CMFE (100, 200 and 400 mg kg^−1^, b.w., p.o.) and SULF (100 mg kg^−1^, b.w., p.o.) or distilled water, and challenged with a single anal administration of AA (300 mg kg^−1^, b.w.) (3%, v/v, 5 mL kg^−1^, b.w.) or NaCl (0.9%, 5 mL kg^−1^, b.w.) for 24 h.

Treatment	Control	UC	UC + CMFE‐100	UC + CMFE‐200	UC + CMFE‐400	UC+SULF
Glucose (mmol/l)	6.58 ± 0.50	12.54 ± 1.90***	9.09 ± 0.05**	7.99 ± 0.22**	6.51 ± 0.53**	6.97 ± 0.26**
TC (mmol/L)	1.25 ± 0.21	1.40 ± 0.08	1.36 ± 0.07	1.37 ± 0.05	1.27 ± 0.01	1.26 ± 0.13
TG (mmol/L)	1.06 ± 0.10	1.06 ± 0.25	1.03 ± 0.11	1.05 ± 0.16	1.01 ± 0.04	1.06 ± 0.12
HDL (mmol/L)	0.55 ± 0.01	0.52 ± 0.03	0.54 ± 0.04	0.56 ± 0.01	0.56 ± 0.01	0.55 ± 0.02

*Note:* Data are expressed as means ± SEM, with different groups compared by one‐way ANOVA followed by Tukey's post hoc test (*n* = 5).

*** *p* < 0.001 compared to control group.

** p < 0.001 compared to AA group.

#### 3.3.3 Impact of CMFE and AA on Inflammatory Markers

**FIGURE 3:**
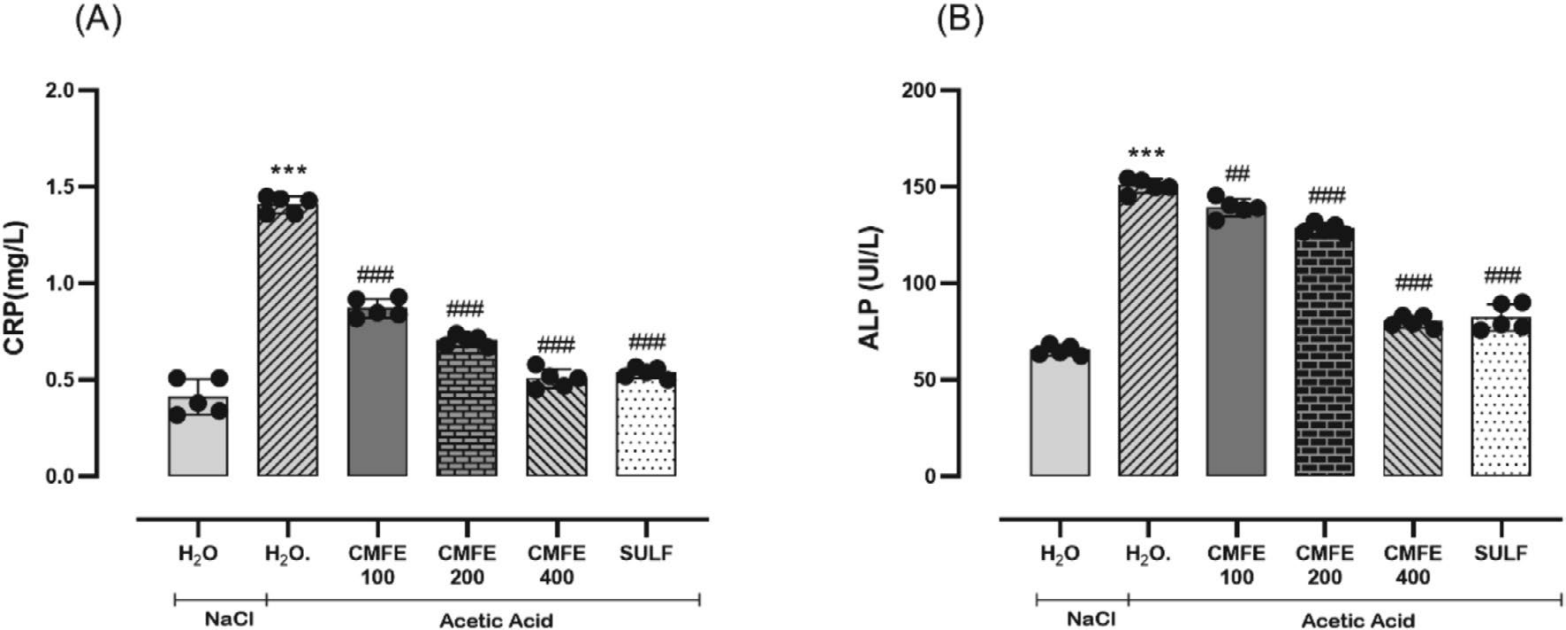
Impact of *Crataegus monogyna* fruits extract (CMFE) and sulfasalazine (SULF) on the changes in inflammatory parameters CRP (A) and ALP (B) induced by acetic acid (AA). Animals were pre‐treated with various doses of the CMFE (100, 200, and 400 mg kg^−1^, b.w., p.o.) and SULF (100 mg kg^−1^, b.w., p.o.) or distilled water, and challenged with a single anal administration of AA (300 mg kg^−1^, b.w.) (3%, v/v, 5 mL kg^−1^, b.w.) or NaCl (0.9%, 5 mL kg^−1^, b.w.) for 24 h. The data are expressed as means ± SEM (*n* = 5), with different groups compared by one‐way ANOVA followed by Tukey's post hoc test. Asterisks denote ***p ≤ 0.001 versus the control group; hash symbols denote ^##^ *p* ≤ 0.01, and ^###^ *p* ≤ 0.001 versus the UC group.

The plasma levels of the inflammatory markers CRP and ALP were significantly higher in the UC group than in the control group, as shown in Figure 3. In comparison to the control group, the CRP level in the UC‐treated group rose significantly from 0.42 ± 0.13 mg/L to 1.40 ± 0.06 mg/L. Following treatment with several dosages of CMFE, the level of this inflammatory marker was significantly and dose‐dependently reduced to 0.52 ± 0.09 mg/L. Furthermore, the results showed that the CRP and ALP levels in the CMFE‐treated groups were almost identical to those in the control group, and even CMFE had a better effect than SULF (Figure 3).

### 3.4 Impact of CMFE and AA on Oxidative Stress Levels

#### 3.4.1 Lipid Peroxidation in the Colon

**FIGURE 4:**
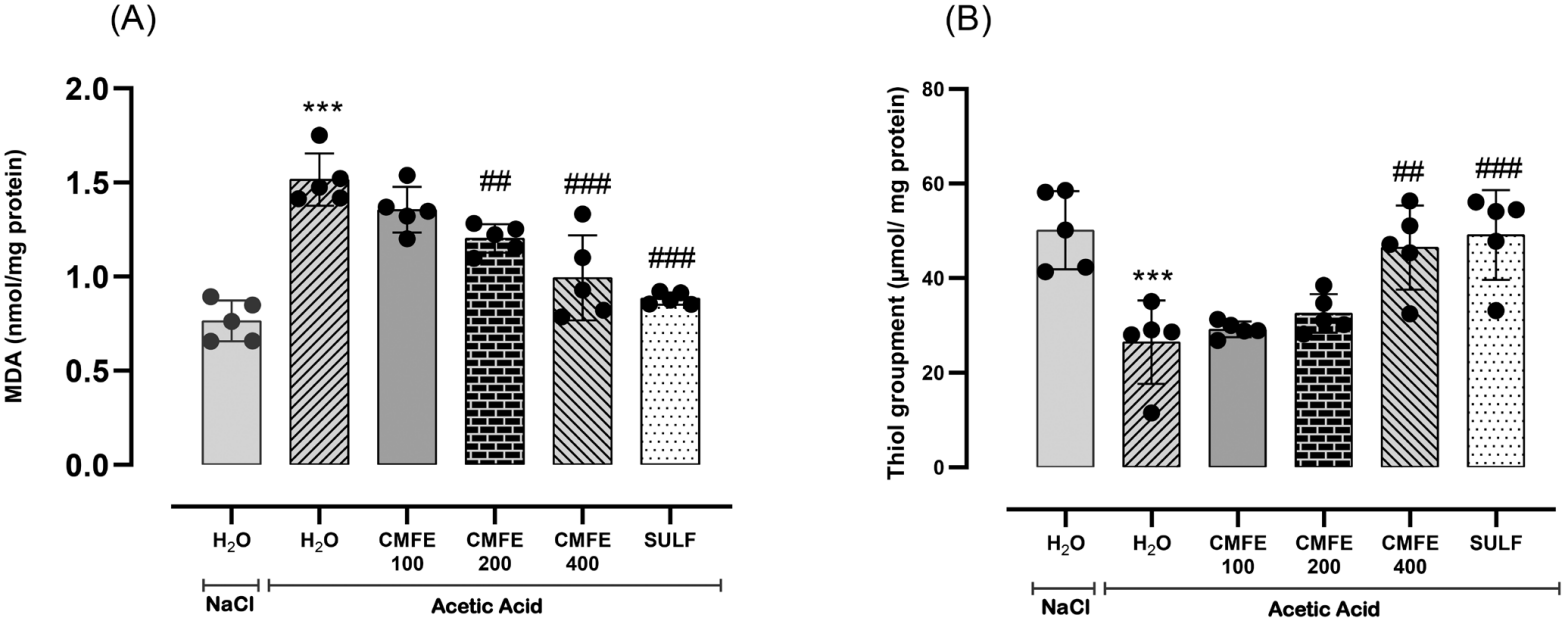
Impact of *Crataegus monogyna* fruits extract (CMFE) and sulfasalazine (SULF) on the levels of MDA (A) and thiol groups (B) levels during acetic acid (AA)‐induced ulcerative colitis. Animals were pre‐treated with various doses of the CMFE (100, 200 and 400 mg kg^−1^, b.w., p.o.) and SULF (100 mg kg^−1^, b.w., p.o.) or distilled water, and challenged with a single anal administration of AA (300 mg kg^−1^, b.w.) (3%, v/v, 5 mL kg^−1^, b.w.) or NaCl (0.9%, 5 mL kg^−1^, b.w.) for 24 h. The data are expressed as means ± SEM (*n* = 5), with different groups compared by one‐way ANOVA followed by Tukey's post hoc test. Asterisks denote ***p ≤ 0.001 versus the control group; hash symbols denote ^##^ *p* ≤ 0.01, and ^###^ *p* ≤ 0.001 versus the UC group.

The administration of AA significantly raised colonic lipid peroxidation levels in the UC group, but pre‐treatment with CMFE and sulfasalazine significantly decreased them in a dose‐dependent manner (Figure 4A). This was done to examine the role of oxidative stress in the anti‐ulcerogenic effect of CMFE.

#### 3.4.2 Level of Colonic Thiol Groups

We showed in Figure 4B that intrarectal AA injection significantly decreased colonic (‐SH) groups, whereas pre‐treatment with sulfasalazine or CMFE (400 mg/kg, BW) significantly prevented this depletion.w.

#### 3.4.3 Activity of Colonic Antioxidant Enzymes

**FIGURE 5:**
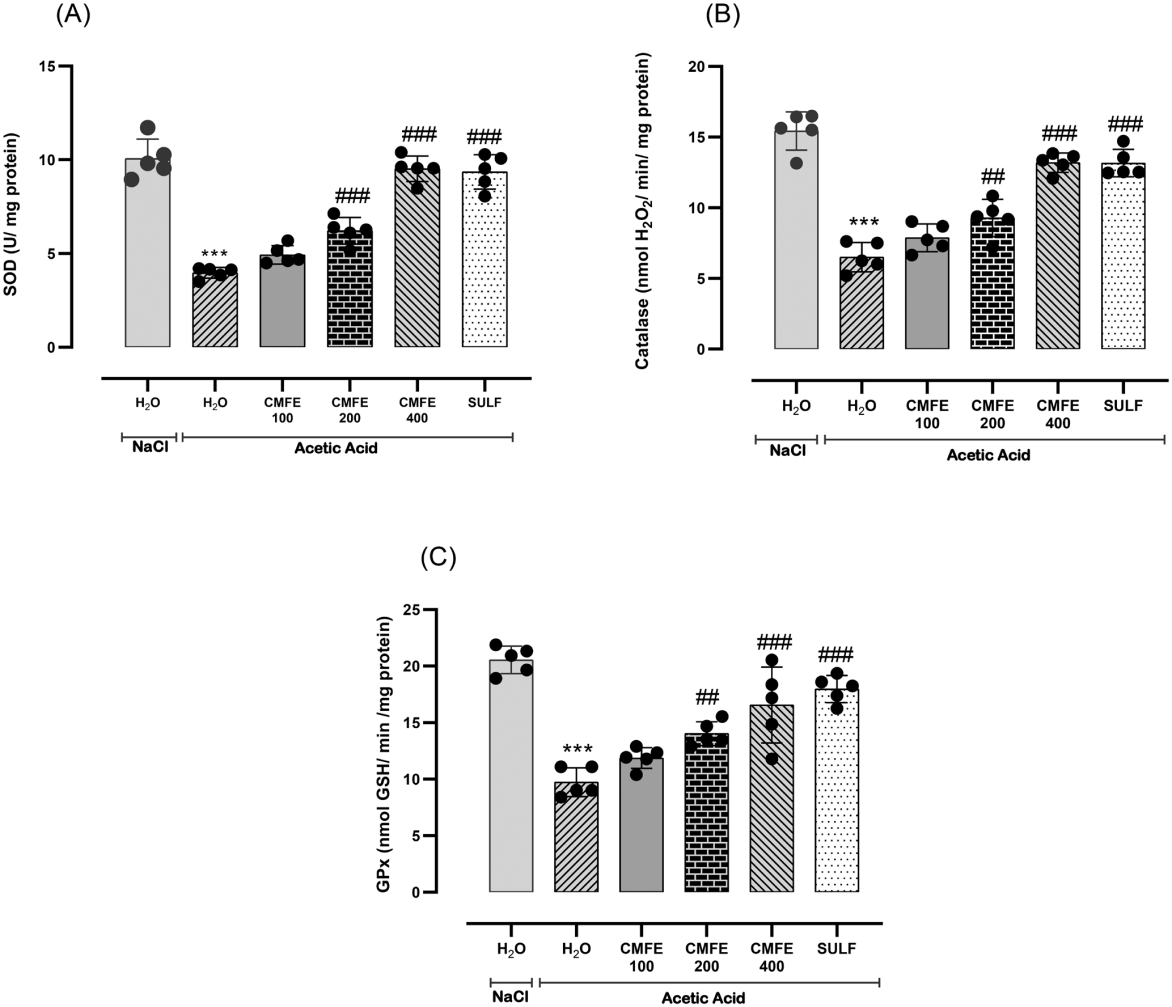
Impact of *Crataegus monogyna* fruits extract (CMFE) and sulfasalazine (SULF) on colonic mucosal antioxidant enzyme activities: SOD (A), CAT (B), and GPx (C) in ulcerative colitis induced by acetic acid (AA). Animals were pre‐treated with various doses of the CMFE (100, 200, and 400 mg kg^−1^, b.w., p.o.) and SULF (100 mg kg^−1^, b.w., p.o.) or distilled water, and challenged with a single anal administration of AA (300 mg kg^−1^, b.w.) (3%, v/v, 5 mL kg^−1^, b.w.) or NaCl (0.9%, 5 mL kg^−1^, b.w.) for 24 h. The data are expressed as means ± SEM (*n* = 5), with different groups compared by one‐way ANOVA followed by Tukey's post hoc test. Asterisks denote ***p ≤ 0.001 versus the control group; hash symbols denote ^##^ *p* ≤ 0.01, and ^###^ *p* ≤ 0.001 versus the UC group.

AA (3%, v/v, 5 mL kg_1, b.w.) significantly decreased the activities of SOD (A), CAT (B), and GPx (C) in the colonic mucosa of AA‐intoxicated animals as compared to the controls (Figure 5). Pretreatment with CMFE (100, 200, and 400 mg/Kg, b.w.) or sulfasalazine (100 mg/Kg, b.w.) over a ten‐day period, however, significantly mitigated this depletion in a dose‐dependent manner. The two highest doses, 200 and 400 mg/kg, b.w. showed significant effects (*p* < 0.01) compared to the colitis group. More significantly, as compared to the reference chemical, sulfasalazine, CMFE had a stronger impact on superoxyde dismutase and catalase activity at a high dose (400 mg/kg bw, po).

### 3.5 Microscopical Assessment of Colonic Lesions: Histopathological Examination

**FIGURE 6:**
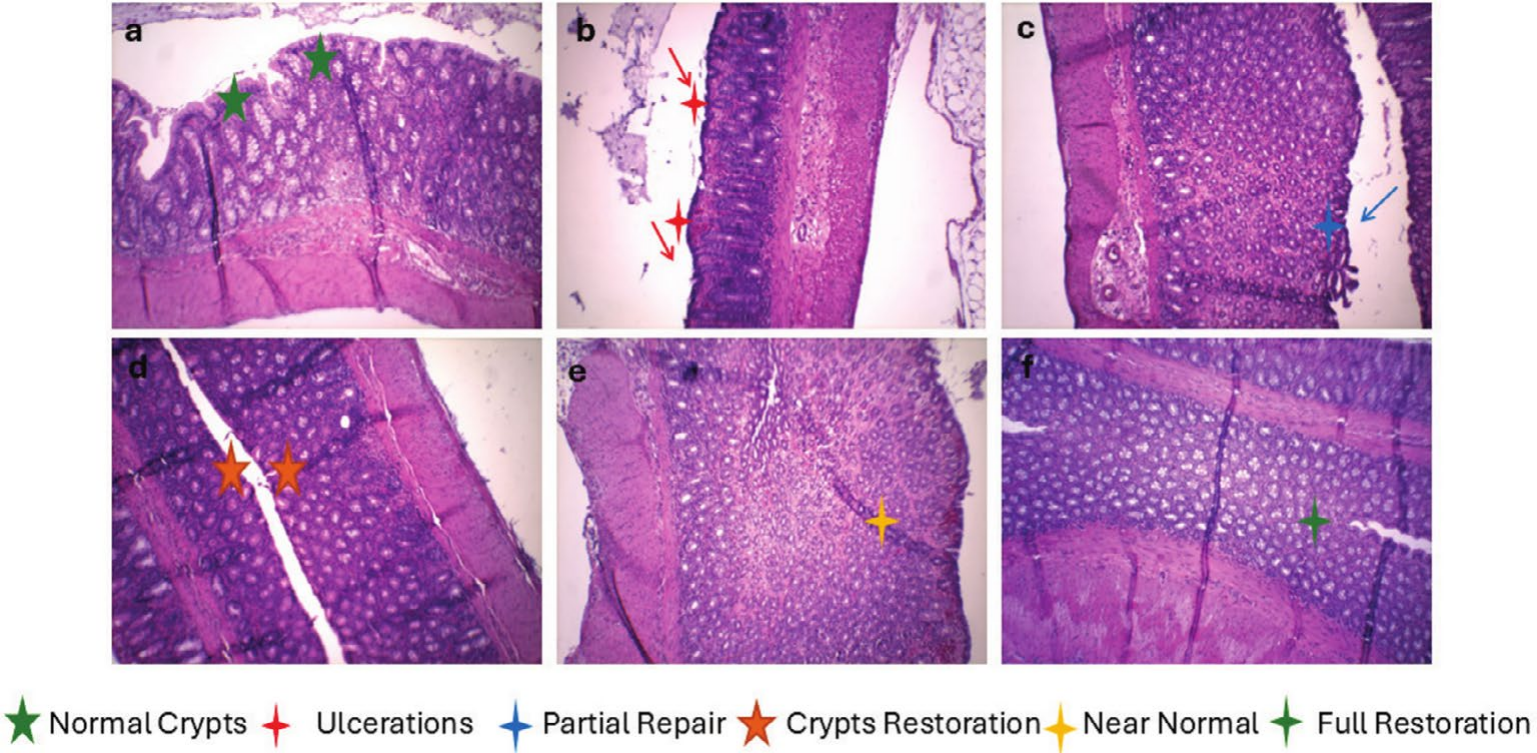
The preventive effects of *Crataegus monogyna fru*its extract (CMFE) and sulfasalazine (SULF) in ulcerative colitis caused by acetic acid (AA) are demonstrated by colon histology. Animals were pre‐treated with various doses of the CMFE (100 mg kg^−1^, 200 mg kg^−1^, and 400 mg kg^−1^, b.w., p. o.) and SULF (100 mg kg^−1^, b.w., p. o.) or distilled water, and challenged with a single anal administration of AA (300 mg kg^−1^, b.w.) (3%, v/v, 5 mL kg^−1^, b.w.) or NaCl (0.9%, 5 mL kg^−1^, b.w.) for 24 h. The histopathological modifications in the slide portions of colon tissue were analyzed by staining with H&E followed by monitoring at 10×. (a) Control group, (b) AA group, (c) AA + CMFE (100 mg kg^−1^, b.w., p. o.), (d) AA + CMFE (200 mg kg^−1^, b.w., p. o.), (e) AA + CMFE (400 mg kg^−1^, b.w., p. o.), (f) AA + SULF.

Histological analysis revealed distinct differences among the experimental groups (Figure 6). In the control group, the colonic mucosa exhibited an intact structure, with well‐organized crypts and normal architecture, and no signs of inflammation, ulceration, or tissue damage were observed. In contrast, the acetic acid (AA) group displayed severe colitis characterized by significant mucosal destruction, prominent ulcerations, crypt disorganization, and extensive inflammatory cell infiltration. Treatment with CMFE at 100 mg/kg showed moderate improvement compared to the AA group, with partial crypt reorganization; however, residual lesions and inflammation persisted. At 200 mg/kg, CMFE demonstrated a more pronounced reduction in mucosal damage, with substantial crypt restoration and diminished signs of inflammation. The most notable effects were observed at 400 mg/kg, where the crypt architecture appeared almost completely restored, with minimal inflammatory infiltration and resolution of most lesions, resembling a near‐normal state. These findings highlight a dose‐dependent efficacy of CMFE in mitigating acetic acid‐induced colitis. Sulfasalazine, the reference drug, exhibited similar results to the CMFE 400 mg/kg group, with fully organized crypts and an almost complete absence of inflammation or mucosal damage, comparable to the control group. These observations were supported by the histological scoring data (Table 5), where AA significantly increased the histological score compared to the control group. CMFE treatment reduced the histological score in a dose‐dependent manner, with significant improvements observed at 200 mg/kg. Similarly, sulfasalazine markedly decreased the histological score, confirming its protective effect (Figure 1).

**TABLE 5:** Effects of *Crataegus monogyna* fruits extract (CMFE) and sulfasalazine (SULF) on acetic acid (AA)‐induced histological changes in colon tissue. Animals were pre‐treated with various doses of the CMFE (100, 200 and 400 mg kg^−1^, b.w., p.o.) and SULF (100 mg kg^−1^, b.w., p.o.) or distilled water, and challenged with a single anal administration of AA (300 mg kg^−1^, b.w.) (3%, v/v, 5 mL kg^−1^, b.w.) or NaCl (0.9%, 5 mL kg^−1^, b.w.) for 24 h.

Treatment	Histological score (Median, range)
Control	0 (0–0)
UC	8 (6–10)*
UC + CMFE 100	7 (7–10)
UC + CMFE 200	6 (4–7)^#^
UC + CMFE 400	3 (3–4)***
UC + SULF	3 (3–4)***

*Note:* Data are presented as median (range); statistical significance was determined by Kruskal–Wallis test followed by Dunn's post hoc test.

* *p* ≤ 0.05 versus control.

^#^ *p* ≤ 0.05.

*** *p* ≤ 0.001 versus UC.

## 4 Discussion

In the present work, we examined the effects of CMFE pre‐treatment on AA‐induced colitis in rats. First, we demonstrated In vivo that acetic acid given acutely anally at a rate of 3% v/v, 5 mL kg^−1^, b.w., clearly resulted in severe ulceration of the colon mucosa. In parallel these morphological changes, the distal colon's hydrated weight rises, which is thought to be a sensitive and accurate indicator of the severity and extent of the inflammatory response (Balogun et al. 2016). The similar outcome was seen in previous studies that employed AA to induce colitis (Hajji et al. 2018; Jedidi et al. 2021; Sammari et al. 2024). Acetic acid is introduced into the colon via the rectum, leading to epithelial damage and apoptosis. This model is useful for simulating the inflammatory responses observed in diseases like ulcerative colitis. It is often used to study the role of immune cells, oxidative stress, and inflammatory pathways in IBD, and it helps in evaluating potential therapeutic agents that could modulate these processes (Muro et al. 2024). Body weight was monitored throughout the experimental period to ensure animal welfare and to assess the general condition of the animals. However, given the acute and short‐term nature of the model, no significant differences were observed between groups, which is consistent with previous reports in similar experimental conditions.

(Ozsoy et al. 2018).

It is crucial to remember that the acetic acid model results in colon ulceration, which leads to distinctive histological alterations include oedematous tissue, ulcer formation, and the emergence of dark red bands in the distal colon (Shahid et al. 2022). These features, which include more or less profound lesions and irritation of the intestinal mucosa, are typical of human colitis (Hartmann et al. 2012). By releasing protons into the intracellular space, acetic acid creates an acidic environment that damages cells in a number of ways (Somani et al. 2014). According to the histological examination of colonic sections, AA caused significant colon inflammation, as evidenced by necrosis, oedema, loss of mucosal integrity, and epithelial congestion, as compared to the control group. When acetic acid causes inflammation, immune cells particularly neutrophils are drawn to the area of damage (Arafa et al. 2020). In several research, rats were given AA as a single dose to induce experimental colitis, yet this effect has been observed (Adakudugu et al. 2020; Elbaz et al. 2023). Mucosal macrophage activation results in the release of several pro‐inflammatory cytokines, including TNF‐α, IL‐1β, IL‐6, and IL‐12. These cytokines increase the inflammatory response, introducing more immune cells to the damage site and keeping the inflammatory cycle going (Zhang et al. 2020). In contrast, all of the macroscopic and histological abnormalities brought on by AA were averted by pre‐treatment with CMFE (100, 200, and 400 mg/Kg, b.w., p.o.) or sulfasalazine (100 mg kg, b.w., p.o.). On the other hand, the quantity of phenolic compounds, which are well known for their anti‐inflammatory and antioxidant properties, is partially linked to the cytoprotection that CMFE provides. CMFE‐treated animals showed a significant dose‐dependent reduction in wet colon weight compared with the colitis group. In fact, CMFE contains phenolic compounds such as syringic acid (Wahabi et al. 2024), which is known as a powerful natural antioxidant that is effective in reducing oxidative stress. In addition, according to Somade et al. (2023), syringic acid has demonstrated anti‐inflammatory effects through the modulation of several genes implicated in inflammation (Somade et al. 2023). Myricetin, another CMFE flavonoid compound, which has also been shown to modify inflammatory skin diseases by suppressing pro‐inflammatory mediators. A separate study showed that myricetin could inhibit NF‐κB, IL‐6, IL‐12, NO, iNOS, TNF‐α, modulate NF‐κB DNA binding activity and NF‐κB p65 subunit degradation, STAT1 phosphorylation in RAW264.7 macrophages stimulated with LPS and IFN‐β (Cho et al. 2016; Latief et al. 2015). For many years, sulfasalazine has been an essential component of treatment for IBD (Helieh S Oz et al. 2013). A drug that is poorly absorbed in the stomach and small intestine, sulfasalazine is formed of 5‐aminosalicylic acid (5‐ASA) and sulfapyridine joined by an azo bond (Helieh S. Oz and Ebersole 2008). It has a metal chelating activity that lowers oxidative stress and functions as an antioxidant against produced ROS and NOS. Furthermore, Sulfasalazine triggers T cell death, inhibits the nuclear factor kappa B (NF‐kB) transcription pathway for pro‐inflammatory cytokines and inflammatory intermediates cyclooxygenase/lipoxygenase, and activates the peroxisome proliferator‐activated receptor (Liptay et al. 2002; Rousseaux et al. 2005).

We have also studied the role of oxidative stress in the pathophysiology of UC. We demonstrated that the colonic mucosa underwent oxidative stress when acetic acid was taken orally, as seen by increased lipoperoxidation, a decrease of non‐enzymatic antioxidants such thiol groupment, and reduced activity of antioxidant enzymes including SOD, CAT, and GPx. These results fully corroborate previous research showing that oxidative balance disruption is associated with acetic acid‐induced colitis (Akgun et al. 2005; El‐Akabawy and El‐Sherif 2019). Overloaded reaction oxygen species in colitis produce oxidative stress and change the redox balance in the gut mucosa, which accelerates the development of colon disease (Balmus et al. 2016). The activity of infiltrating leukocytes, especially neutrophils and macrophages, within the colon increases as Ulcerative Colitis (UC) worsens. Oxidative stress is exacerbated by the increased synthesis of pro‐oxidant molecules brought on by this heightened immunological response. Myeloperoxidase (MPO), an enzyme secreted by neutrophils, is also enhanced in response to the inflammatory cytokines linked to ulcerative colitis. MPO activity is essential for the production of reactive oxygen species (ROS), which exacerbates inflammation and tissue damage in the colon (Khan et al. 2018). Yet, by lowering ROS generation and increasing the colon's antioxidant defenses, therapy with various doses of CMFE reduced the oxidative stress induced by AA in the colon. The In vitro antioxidant potential of the *Crataegus monogyna* aqueous extract was rigorously evaluated using two complementary assays, namely DPPH (2,2‐diphenyl‐1‐picrylhydrazyl) and ABTS (2,2′‐azino‐bis 3‐ethylbenzothiazoline‐6‐sulfonic acid) (Wahabi et al. 2023). The extract demonstrated a potent and dose‐dependent capacity to scavenge both DPPH and ABTS free radicals, indicating a significant electron‐donating ability and a broad‐spectrum neutralizing effect (Wahabi et al. 2023). These findings confirm the intrinsic antioxidant properties of the hawthorn fruit extract, which are attributable to its rich profile of bioactive phytochemicals.

Indeed, CMFE is rich in quercetin, myricetin, and catechin, flavonoids known for their ability to trap reactive oxygen species and serve as antioxidants (Justino 2017). Flavonoids' hydroxyl groups or extremely reactive substituents are primarily responsible for their antioxidant qualities or free radical scavenging capabilities (Almodaifer et al. 2017).

As well, we demonstrated that AA intoxication caused an excess of colonic ionisable calcium, and that CMFE treatment returned calcium homeostasis to baseline levels. It is commonly known that oxidative stress can cause calcium from the external environment as well as the sarcoplasmic reticulum (SR) or endoplasmic reticulum (ER) to enter the cytoplasm through their respective channels and the cell membrane (Ermak and Davies 2002). Part of the protective effect of CMFE against UC treatment may be due to the maintenance of balanced intracellular calcium flow. A similar mechanism has previously been established in UC caused by AA established (Hajji et al. 2018). Additionally, we found that AA increased plasma levels of inflammatory markers like phosphatase alkaline activity and C‐reactive protein content. However, pretreatment with sulfasalazine and CMFE reduced the inflammatory markers in a dose‐dependent way. Although plasma free iron and calcium were assessed as systemic biochemical indicators reflecting the broader inflammatory and metabolic alterations associated with acetic acid‐induced colitis, they should not be considered direct markers of intestinal mucosal healing, and their changes are interpreted here only as supportive evidence of the overall physiological impact of CMFE.

Intestinal alkaline phosphatase (IAP) has an important role in gut mucosal defense. Expression of IAP is known to be affected by prematurity, starvation, and inflammation (Lallès 2014). Although studies have shown that CRP levels increase during infections and inflammatory diseases (Sproston and Ashworth 2018), CRP is mostly produced by IL‐6‐dependent hepatic biosynthesis and is the main downstream mediator of the acute‐phase response to an inflammatory event (Baumeister et al. 2016; Pradhan et al. 2001). The primary function of CRP in inflammation is thought to be related to the complement pathway's activation of the C1q molecule, which causes pathogens to opsonize. While CRP can activate the complement system to start the host defense fluid phase pathways, it can also activate complement and bind to IgG's Fc receptors to start cell‐mediated pathways (Pradhan et al. 2001). When CRP binds to Fc receptors, pro‐inflammatory cytokines are released as a result of the interaction (Du Clos 2000).

In the current study, we also showed that AA‐induced colitis was linked to a disruption in the features of the plasma lipid profile, as indicated by higher LDL cholesterol levels. Subacute pretreatment with SULF and CMFE, however, has considerably reduced these conditions. *Crataegus monogyna* fruits have been shown to have a hypolipidemic effect on healthy rats (Wahabi et al. 2023). Future studies should focus on isolating and testing individual bioactive compounds from CMFE (e.g., myricetin, quercetin) to elucidate their specific roles in coloprotection. Additionally, investigating the impact of CMFE on gut microbiota composition, tight junction proteins, and specific inflammatory pathways would provide deeper mechanistic insights. Long‐term In vivo studies and clinical trials are also warranted to assess the efficacy and safety of CMFE in human IBD.

This study has several limitations. First, the acetic acid model, while widely used, does not fully replicate the chronic and immune‐mediated nature of human ulcerative colitis. Second, only male rats were used to control for hormonal variability; future studies should include both sexes to evaluate potential gender differences. Third, the exact bioactive compounds responsible for the observed effects remain to be isolated and tested individually. Finally, the study focused on preventive pretreatment; the therapeutic potential of CMFE in established colitis remains to be investigated.

## 5 Conclusion

Overall, our results support the benefits of *C. monogyna* fruits and their potential as a preventive treatment for gastrointestinal disorders, as demonstrated in the case of ulcerative colitis. In conclusion, CMFE was shown to offer protection against acetic acid‐induced ulcerative colitis through histological and macroscopic improvements, consistent with its demonstrated antioxidant properties and previously reported anti‐inflammatory potential.

## Author Contributions

**Soumaya Wahabi:** conceptualization, methodology, data curation, writing – original draft, investigation, visualization, formal analysis, software. **Khaoula Gharbi:** methodology. **Slimen Selmi:** writing – review and editing, validation. **Hichem Sebai:** supervision, writing – review and editing. **Dhekra Grami:** methodology.

## Funding

The authors have nothing to report.

## Disclosure

Statement of Human and Animal Rights: All procedures in this study were conducted in accordance with the * NIH guidance and the local ethics council of Tunis University * approved protocols.

## Ethics Statement

Ethical approval to report this case was obtained from *Comité d'Ethique Bio‐medicale (CEBM)’ (JORT472001) of the ‘Institut Pasteur de Tunis*.

## Consent

The authors have nothing to report.

## Conflicts of Interest

The authors declare no conflicts of interest.

## Data Availability

The data that support the findings of this study are available on request from the corresponding author. The data are not publicly available due to privacy or ethical restrictions.
